# Intraocular Penetration of a vNAR: In Vivo and In Vitro VEGF_165_ Neutralization

**DOI:** 10.3390/md16040113

**Published:** 2018-03-31

**Authors:** Tanya A. Camacho-Villegas, María Teresa Mata-González, Walter García-Ubbelohd, Linda Núñez-García, Carolina Elosua, Jorge F. Paniagua-Solis, Alexei F. Licea-Navarro

**Affiliations:** 1 CONACYT-Medical and Pharmaceutical Biotechnology, Centro de Investigación y Asistencia en Tecnología y Diseño del Estado de Jalisco (CIATEJ), Guadalajara, Jalisco, C.P. 44270, Mexico; tcamacho@ciatej.mx; 2 Biomedical Innovation Department, Centro de Investigación Científica y Educación Superior de Ensenada, (CICESE), Ensenada, Baja California, C.P. 22860, Mexico; 3 Research and Development Department, Laboratorios Silanes S.A. de C.V., Ciudad de México, C. P. 03100, Mexico; tmata@silanes.com.mx (M.T.M.-G.); wgarcia@silanes.com.mx (W.G.-U.); lnunez@silanes.com.mx (L.N.-G.); jpaniagua@laboratoriosilanes.es (J.F.P.-S.); 4 Research and Development Department, Teraclón IDF S.L., Calle de Santiago Grisolía, Tres Cantos, 28020 Madrid, Spain; c.elosua@teraclon.com

**Keywords:** vNAR, single chain binding domain, VEGF_165_, intraocular penetration, *Heterodontus fransisci*, horn shark, age-related macular degeneration, diabetic retinopathy

## Abstract

Variable new antigen receptor domain (vNAR) antibodies are novel, naturally occurring antibodies that can be isolated from naïve, immune or synthetic shark libraries. These molecules are very interesting to the biotechnology and pharmaceutical industries because of their unique characteristics related to size and tissue penetrability. There have been some approved anti-angiogenic therapies for ophthalmic conditions, not related to vNAR. This includes biologics and chimeric proteins that neutralize vascular endothelial growth factor (VEGF)_165_, which are injected intravitreal, causing discomfort and increasing the possibility of infection. In this paper, we present a vNAR antibody against human recombinant VEGF_165_ (rhVEGF_165_) that was isolated from an immunized *Heterodontus francisci* shark. A vNAR called V13, neutralizes VEGF_165_ cytokine starting at 75 μg/mL in an in vitro assay based on co-culture of normal human dermal fibroblasts (NHDFs) and green fluorescence protein (GFP)-labeled human umbilical vein endothelial cells (HUVECs) cells. In the oxygen-induced retinopathy model in C57BL/6:Hsd mice, we demonstrate an endothelial cell count decrease. Further, we demonstrate the intraocular penetration after topical administration of 0.1 μg/mL of vNAR V13 by its detection in aqueous humor in New Zealand rabbits with healthy eyes after 3 h of application. These findings demonstrate the potential of topical application of vNAR V13 as a possible new drug candidate for vascular eye diseases.

## 1. Introduction

In the group of Vascular endothelial growth factor isoforms, VEGF type A (VEGFA, which include VEGF_165_, 45 kDa) has been characterized as a mitogen that promote angiogenesis for vascular endothelial cells from arteries, veins, and lymphatic tissue [1]. In events of hypoxia VEGF_165_ is overproduced, turning on pathological angiogenesis in a variety of eye diseases, such as age-related macular degeneration (AMD), diabetic retinopathy (DR), diabetic macular edema (DME), neovascular glaucoma, and retinal vein occlusion (RVO) [2,3]. During rapid, uncontrolled ocular angiogenesis, new vessels are formed from the existing vascular tree, leading to vascular fragility and thus hemorrhage and accumulation of fluids or proteins that affect various structures in the eye, including retina, choroid, and cornea [4]. This pathological angiogenesis process can cause vision loss or blindness [5]. Different pathways such as PKCβ, ERK1/2, and Rho/Rho kinase could upregulate the expression of VEGFA in diabetic retinopathy [6]. Additionally, the overexpression of pro-inflammatory molecules such as ICAM-1, TNFα, PGs (prostaglandins), PLA2 (phospholipases 2), and others, is a pro-inflammatory component in DR pathogenesis [7]. Some of those pro-inflammatory molecules have been investigated as therapeutic targets [8].

In the past 15 years, many drugs that inhibit VEGF_165_ activity have been developed and used in therapy [9,10]. Additionally, ocular drug delivery has been a major challenge due to the unique anatomy and physiology of the eye, requiring biotechnological innovations in the development of therapeutic drugs [11,12].

Such advances, based on monoclonal antibodies or chimeric proteins, include ranibizumab a recombinant humanized Fab fragment (48 kDa) that inhibits all isoforms of VEGFA (including VEGF_165_). Ranibizumab is administered by intravitreal injection, but other routes are being analyzed in preclinical assays, such as subconjunctival and intracameral injection. Ranibizumab stabilizes and improves vision in over 90% of patients [13].

Bevacizumab is a humanized recombinant antibody that binds all isoforms of human VEGFA (including VEGF_165_) and was approved in 2004 by the FDA only for glioblastoma (GMB), metastatic colorectal cancer (mCRC), non-small-cell lung cancer (NSCLC), and metastatic kidney cancer (mRCC). Bevacizumab has had good results in other diseases, nevertheless to date, it has not been approved for ocular use, and therefore more research is still going in the field [14]. Although many preclinical studies have reported significant evidence that supports the use of bevacizumab in patients [15,16], inflammation was detected after intraocular injection range from 0.3% [17] to 1.3% [18] and this incidence was higher (69%) in some patients due to the presence of endotoxins in one bevacizumab product lot [19]. Before obtaining FDA approval, more clinical trials need to be done to determine the efficacy, optimal dosage, safety, and side effects of topical bevacizumab in corneal epithelium [20].

Aflibercept is a fully humanized soluble chimeric fusion protein in which the extracellular domains of VEGF_165_ receptor I and II are linked to the Fc portion of human IgG1. Aflibercept has been approved for neovascular AMD and is administered monthly by intravitreal injection [21].

Although these drugs are considered disease modifying drugs and are effective against AMD and related eye diseases such as DME, choroidal neovascularization (CNV), RVO, the repeated intravitreal administration can be associated with significant anxiety and discomfort, and carries the risk of complications such as ocular pain, infection, hemorrhage, and retinal detachment [10]. These procedures require a specialist and are expensive, necessitating novel drugs that can be applied more simply and safely in the eyes.

In this regard, a new promising approach is the use of heavy chain antibodies called IgNARs, which are found in the immunoglobulin repertoire of sharks. These antibodies have a highly soluble single antigen-binding variable domain (vNAR) with a long complementarity-determining region 3 (CDR3) (10–26 aa) [22]. The vNARs which can be obtained from naïve, immunized or synthetic shark libraries, have low molecular weight (12–15 kDa) and can resist the low pH present in the human gastrointestinal tract and relatively high urea levels, as well as being thermostable [23]. The vNARs, are more suitable for recombinant expression, tissue penetration, and can bind to cryptic targets in comparison with Fab or scFv fragments [22,23,24,25]. Müller [26] improved the pharmacokinetic properties (half-life) of a vNAR by fusing it to human serum albumin (HSA) and reported no immunogenicity, supporting the potential of multifunctional therapies. Previously in our group, we isolated a vNAR that can recognize the recombinant human TNFα cytokine. Moreover, the vNAR demonstrated neutralization capacity in an endotoxic shock murine model [27]. vNARs are small molecules with high stability and solubility, good pH resistance, thermostability, and tissue penetration, all of which are advantageous and render them excellent immunotherapeutic candidates [28,29,30].

In this study, we isolated and characterized a neutralizing VEGF_165_ vNAR. By co-culture assay of normal human dermal fibroblasts (NHDF) and GFP-labeled HUVECs cells after vNAR treatment, we observed a significant reduction in tube length, tube area, and branch point formation of the microvascularity. We detected vNAR V13 in aqueous humor in healthy rabbit eyes after topical administration. These data indicate that the topical application of the V13 vNAR is a promising candidate for ophthalmological disorders that are associated with neovascularization.

## 2. Results

### 2.1. vNAR Phage Display and Sequence

**Figure 1:**
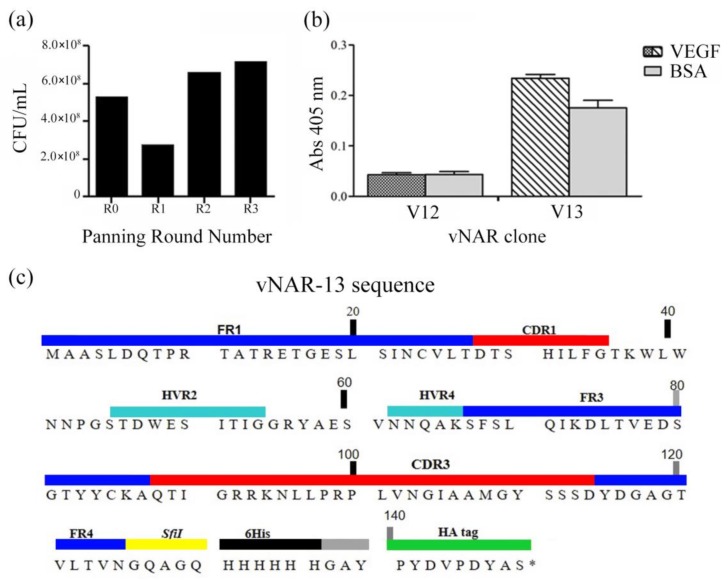
Phage display rounds from vNAR immune library isolated from *Heterodontus francisci* shark against rhVEGF_165_. (**a**) After 3 panning rounds using an immune library, an increase in bacteriophage displaying a specific vNAR was observed; (**b**) two clones expressing a vNAR were analyzed to verify specific recognition ability. The V12 clone has the same recognition ability of rhVEGF_165_ or BSA. The V13 clone has a better recognition of VEGF_165_ compared with BSA; (**c**) the V13 protein sequence showing a long CDR3 (27 aa) with neutralization capacity against rhVEGF_165_. CFU: colony-forming unit.

After three rounds of panning, an increase of specific phages against the antigen (CFU/mL) was observed in round 3, representing enrichment of the selected phages under applied stringency (Figure 1a). A total of 20 isolated clones were analyzed by PCR, the best clone determined by an enzyme-linked immunosorbent assay (ELISA) was selected for initial protein expression and recognition. Figure 1b shows the selected clone (V13) and an example of a non-active clone (V12). Only the V13 clone was selected for further analysis. V13 has a long CDR3 (27 amino acids) and can be classified as an IV or IIb isotype (Figure 1c) [31,32]. This type of vNAR lacks the non-canonical cysteine residues that are found in other types of vNARs; thus, this paratope is more flexible [33]. The sequence obtained in this work was described and protected in US8496933B2 Patent.

### 2.2. Expression and Purification of vNAR from Inclusion Bodies

**Figure 2:**
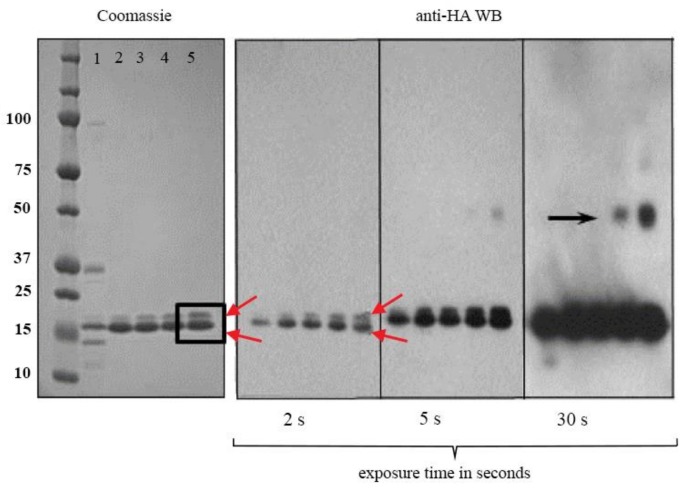
Characterization and analysis of molecular weight of V13. (**a**) SDS-PAGE stained with Coomassie blue showing four batches of purified antibody (lanes 2–5); (**b**) Western blot of the four batches of purified V13, detected using the HA identification tag at different time of exposure. Lanes 2 and 4 correspond to 5 µg of V13 and, lanes 3 and 5 correspond to 10 µg both purified under denaturing conditions. Red arrows indicate double bands of V13 recognized by the anti-HA. Black arrow indicates disulfide bond dimers present in the refolded conformation (represent < 1%). A total of five batches of refolded material was prepared, summing 37 mg of refolded vNAR.

V13 was expressed in *E. coli*, it has a molecular weight of 15.9 kDa (theoretical molecular weight (MW) is 15.95 kDa and 15.928 by mass spectrometry) including the 6-His and hemagglutinin (HA) tags (Figure 2a). We observed that a high-level expression led to the formation of inclusion bodies. V13 was extracted under denaturing conditions and after the refolding protocol, the vNAR was analyzed by Western blot (WB), where it was detected at the expected molecular weight. We also detected multimers at 30 s of exposure time (Figure 2b, black arrow) and two characteristic bands of recombinant proteins expressed in *E. coli* with His tag, these two bands were detected in Coomassie staining and in WB (Figure 2, red arrows). The V13 and additional mass of 16.15 and 16.36 kDa were detected [34]. A 90% vNAR recovery was obtained after the removal of endotoxins. The final endotoxin level of the sample was <0.1 EU/mL.

### 2.3. Angiogenesis Co-Culture Assay

**Figure 3:**
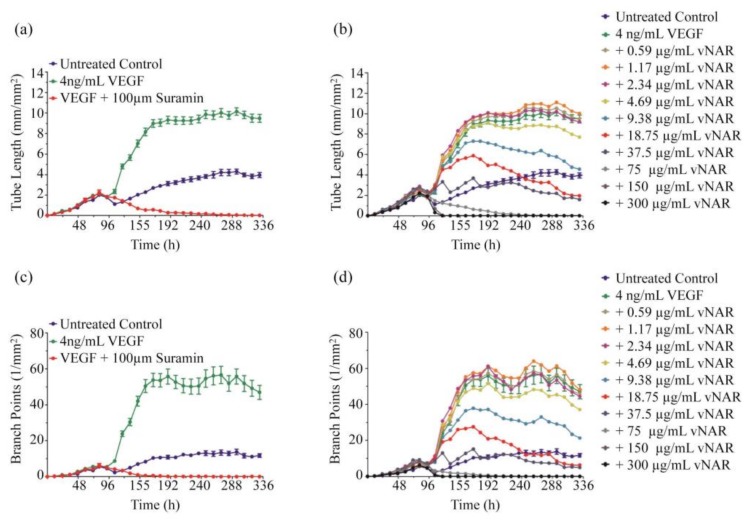
Kinetic responses of V13 in the Essen BioScience Angiogenesis co-culture assay. (**a**) VEGF stimulates tube formation over untreated control as measured by tube length compared to the control containing 100 µM of suramin; (**b**) the V13 inhibit VEGF_165_-driven tube formation in a concentration dependent manner; (**c**) VEGF_165_ stimulates branch point formation over the untreated control as measured by branching (1/mm^2^); (**d**) the V13 inhibit VEGF_165_-driven branching in a concentration dependent manner.

We determined the inhibition kinetic response of V13 by a co-culture assay. In the wells that were treated with 4 ng/mL VEGF_165_, tube length (Figure 3a) and branch formation (Figure 3c) increased compared with those with the same concentration of VEGF_165_ and 100 μM suramin, a potent VEGF_165_ inhibitor [35].

Starting at 9.38 μg/mL (0.58 μM), V13 inhibited tube length and branch point formation in a concentration dependent manner (Figure 3b,d). At 37.5 μg/mL (2.35 μM), V13 nearly completely suppressed these events, reverting levels to untreated control values. For concentrations above 37.5 μg/mL (2.35 μM), total inhibition was observed, similar to the effects of suramin. A significant inhibition was observed with V13 at 75 μg/mL (4.7 μM), after 142 h of incubation. The branch point formation; tube length and tube area also declined at the same time point. At higher concentrations of V13, including 150 and 300 μg/mL (9.4 μM and 18.8 μM respectively), a significant inhibition was reached after 118 h of incubation.

**Figure 4:**
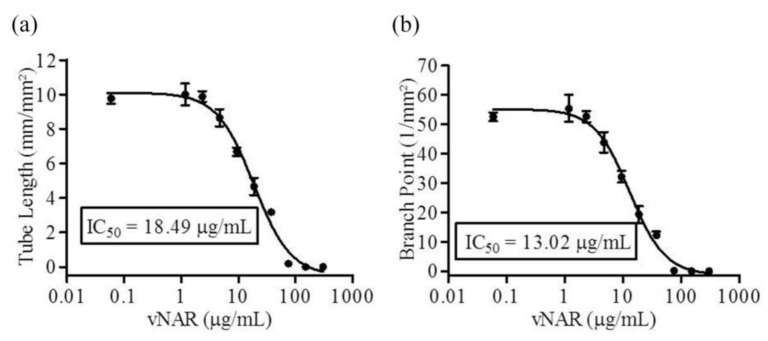
Concentration response analysis for the V13 in angiogenesis co-culture assay. (**a**,**b**), concentration response curves were generated for the tested compound using the non-linear regression model for both tube length (**a**) and branch point (**b**) metrics. The calculated IC_50_ values for tube length was 18.49 µg/mL (1.16 μM) and for branch points of 13.02 µg/mL (817 nM).

By area under the curve (AUC) analysis, VEGF_165_ stimulated extensive tube formation compared with the untreated control. Using the non-linear regression model, V13 had IC_50_ values of 18.49 μg/mL (1.16 μM) for tube length (Figure 4a) and 13.02 μg/mL (817 nM) for branch points (Figure 4b). This analysis shows that V13 inhibits branching and tube formation in a concentration-dependent manner. A Kruskal-Wallis analysis was applied in order to determinate statistical significance. Commercial bevacizumab which is a complete antibody that neutralizes VEGF_165_, has an IC_50_ for tube length of 47.8 μg/mL (320 nM) [36].

### 2.4. Oxygen-Induced Retinopathy (OIR) Model

**Figure 5:**
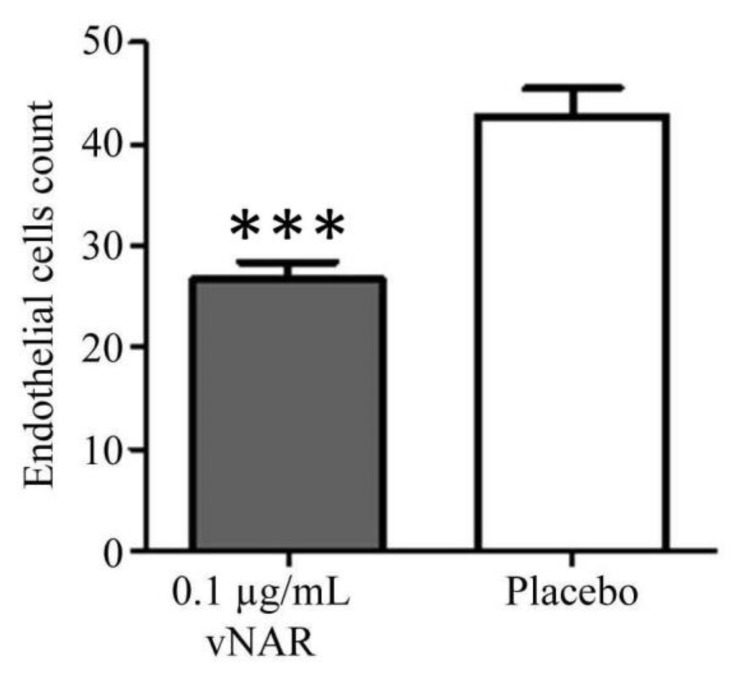
Oxygen-Induced Retinopathy (OIR) assay and protective effect of a dose of V13 compared with placebo against endothelial cell proliferation retina in the OIR model. A significant difference was found in epithelial cell count after topical administration of 0.1 μg/mL of V13. *** indicates *p* < 0.0001 compared to the control. *n* = 20.

The vNARs penetration and neutralization ability in corneal tissue has not been examined extensively. We used an OIR model in mice to demonstrate the protective effects of a 0.1 μg/mL (6.27 nM) dose of V13 compared with placebo against the proliferation of retinal endothelial cells. The difference between both experimental groups (V13 vs. placebo) demonstrated a 30% less proliferation of endothelial cells under the administration scheme, with a *** *p* < 0.0001 obtained by unpaired (two sample) *T*-test (Figure 5). 

### 2.5. Intraocular Penetration

**Figure 6:**
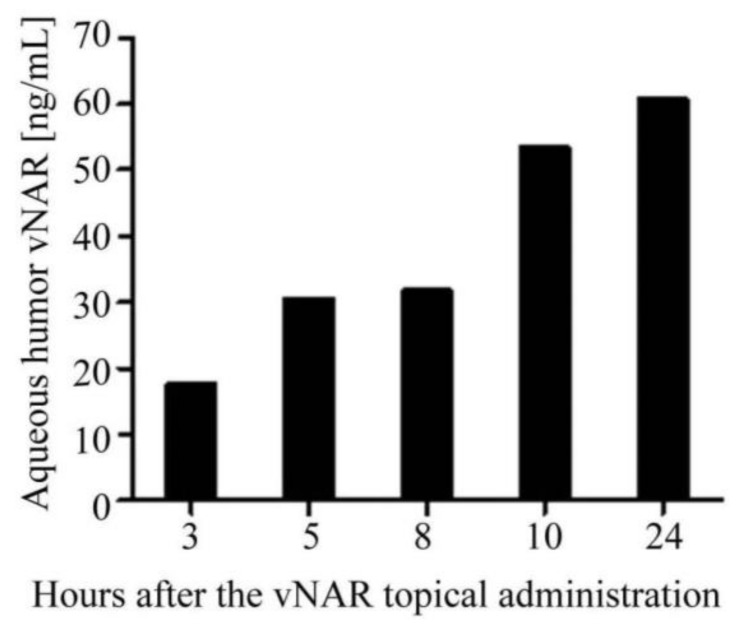
Intraocular penetration of topical V13 administration in rabbits. V13 vNAR (0.1 μg/mL) was administered every 20 min for 10 h. Samples of aqueous humor were collected at various times and measured by ELISA (the results correspond to a single assay).

The OIR model results demonstrated that V13 penetrates and neutralizesthe VEGF_165_ cytokine effect on eyes. As a first approach we evaluated the V13 permeation capability in a healthy rabbit eyes by topical administration. We detected V13 vNAR in aqueous humor after 3 h of treatment, increasing gradually from 20 to 60.68 ng/mL at 24 h (Figure 6). It is well known, that complete antibodies have limited capacity to penetrate corneas with intact epithelium [37].

## 3. Discussion

A shark vNAR (V13) that neutralizes VEGF_165_ was isolated using phage display, the most relevant result of this work was the confirmation that after topical administration, increasing concentrations of V13 could be detected in the aqueous humor of a rabbit eye and that in a mouse model a decrease in proliferation of retinal endothelial cells was detected, with a second indirect demonstration of V13 penetration into eye tissue to perform the biological effect. In our assays, the animals did not show any signs of discomfort. These findings are important and support the use of this unique shark domain as an eye drop for human diseases, such as AMD and DR. The vNAR’s target (VEGF_165_) is one of the main endogenous proangiogenic cytokines.

The introduction of FDA- and EMA-approved anti-VEGF drugs to ophthalmology over the past 12 years has revolutionized the treatment of AMD, macular edema following retinal vein occlusion (RVO), DME, DR, non-proliferative DR (NPDR), proliferative diabetic retinopathy (PDR), and DME [5,38]. However, the anti-VEGF drugs are administered intravitreally [14]. This intravitreal administration is generally well tolerated but has several side effects [39]. Our findings indicate that the V13 vNAR-probably due to size, can penetrate the cornea without injection or abrasion of tissue surface. This characteristic of vNAR, could be an advantage for patient treatment. In the near future, two administrating options could be developed: eye drops, or an intravitreal injection.

Several studies have attempted to determine the appropriate uses for topical immunotherapy [40,41,42,43] with regard to the unique characteristics of the eye. Corneal avascularity [44], dynamics of the aqueous humor [45], and the presence of a cornea with tight junctions, preclude the topical application of a complete antibody because it cannot penetrate beyond the epithelial barrier in healthy corneas. For this reason, several forms of antibody fragments and chimeric proteins have been tested for topical administration. Nevertheless, the vNAR stands as the smallest non-chimeric antibody fragment to recognize and neutralize the biological effect of VEGF_165_ when it is administrated topically on the corneal tissue. In a rabbit animal model, ranibizumab given topically shows low penetrability in the eye, even though this antibody fragment is 48 kDa. In addition, rabbit antibodies against ranibizumab have been detected in rabbit serum after two weeks of the first application, regardless of administration strategy, suggesting a potential immunogenic risk [46]. One of the principal advantage of vNAR, is the low reported immunogenic response [22,26]. We have not found immunogenic response for previous isolated vNAR in our group. However, a further in depth investigation needs to be done.

The properties of an IgNAR should guide the development of new bio-therapeutic drugs that preserve its unique characteristics [25,26,27,29,30]. Recently, Kovaleva [47] reported the selection of a vNAR with high affinity to inducible T-cell co-stimulator ligand (ICOSL). A chimeric vNAR was generated fused with the human Fc fragment. They also demonstrated in a scratched corneal mouse model, a better penetration of the single vNAR compared to the vNAR Fc. This could be relevant to eye uveitis.

In this work, a vNAR was selected against the VEGF_165_ cytokine. It was administered topically in healthy rabbit eyes and detected in aqueous humor after 3 h. In order to reach a clinical application, preclinical tests must be carried out to clearly establish the dosages.

Even when V13 has a lower activity when compared with the reported activity of bevacizumab (1.16 μM and 320 nM respectively), we consider that the use of V13 could be an advantage for the patient. All the intravitreal antibodies for VEGF neutralization, need to be administered once every one or two months, on the other hand, if V13 needs to be administered one or even three times per day, the use of drops is much less aggressive. V13 is a thermo-stable protein, single-dose application plastic vials can be generated, even when the filled temperature needs to be at 50 °C. This is an additional advantage of V13 to gain access to the market.

Nevertheless, we obtained a versatile and efficacious antibody fragment with ocular penetration that can be delivered using an alternate route of administration, such as topical application using eye drops, compared to current methods. To the best of our knowledge, this is the first report that explores this possibility in healthy eyes.

## 4. Materials and Methods

### 4.1. vNAR Fragment Isolation from Immune Library

A male *Heterodontus francisci* shark was immunized iv with 1 μg rhVEGF_165_ (Peprotech (Rocky Hill, NJ, USA), 300-01A) in 1× PBS every 15 days for 20 weeks. The dissection of the spleen, mRNA purification, and library generation were performed as described [48]. For the phage display, 3 rounds of panning were completed against 250 ng/well of rhVEGF_165_ with 5, 10, and 20 wash cycles with 1× PBS/0.5% Tween (PBST-0.5). Positive colonies were selected by PCR with the ompseq and gback primers [49]. Positive PCR clones were grown in LB medium (Sigma (St. Louis, MO, USA), L3022), and plasmids were isolated using commercial kits (Qiagen (Hilden, Germany), 27104). Their sequences were obtained by capillary electrophoresis (Seqxcel, San Diego, CA, USA), analyzed on a CLC DNA Workbench (Qiagen, version 121 7.9.0, Redwood, CA, USA), and compared with internal and external databases using NCBI BLAST.

### 4.2. vNAR Expression and Purification

To express vNARs, the *E. coli* Rosetta(DE3)pLysS cells (Novagen (Madison, WI, USA), 70954) were electroporated with pGW8 plasmid (Genway, San Diego, CA, USA), with the cloned gene into the NdeI-XhoI sites (Promega (Madison, WI, USA), R6801 and R6161). The culture was incubated at 37 °C overnight in liquid LB medium (Sigma, L3022) that contained 100 μg/mL ampicillin (Sigma, 10835242001) at 200 rpm; 6 L of LB medium was inoculated with 1/10 of the overnight culture until an OD600nm value of 0.75 at 37 °C and 200 rpm. The culture was induced with 1 mM IPTG (Sigma, 129 I6758), and cells were grown for an additional 3 h. The bacterial cells were centrifuged at 3500 rpm for 20 min at 4 °C and then, suspended in 100 mL H_2_Odd water, and stored overnight at −20 °C. 

The frozen bacterial pellet was thawed in water at room temperature, and 50 mM Tris-HCl (Sigma, PHG0002) pH 8, 1 μg/mL DNAase (Sigma, DN25), 0.1 M NaCl (Sigma, 793566), and 0.01% Triton (Sigma, T8787) were added to a final volume of 300 mL. The bacterial lysate was incubated at room temperature for 15 min and sonicated (Misonix (Farmingdale, NY, USA), XL-2000) at 11 kHz for 1 min with 30 s of rest in a water bath until it was clarified. The lysate was centrifuged at 14,000 rpm for 30 min at 4 °C, and the supernatant was discarded. The resulting pellet, containing the inclusion bodies, was washed 5 times at room temperature with 0.5 mL 1× PBS by vigorous shaking and vortexing. Each wash step was followed by centrifugation at 14,000× *g* rpm for 10 min at 10 °C, the washes were repeated twice. Inclusion bodies were vigorously suspended in 25 mL of a freshly prepared solution of 8 M urea and 50 mM Tris-HCl, pH 8 and incubated for 1–2 h at room temperature, with occasional stirring or vortexing. Solubilized inclusion bodies were centrifuged at 14,000 rpm for 15 min at 15 °C, and the supernatant was collected.

To determine which clones had the ability to recognize VEGF_165_, an ELISA was performed where 250 ng/50 μL of the rhVEGF_165_ cytokine was placed in well per triplicate, incubated for 1 h at 37 °C and blocked with 150 μL of 3% BSA/1× PBS, incubating 1 h at 37 °C. Afterwards, the liquid was decanted and 50 μL of vNAR protein extracted by inclusion bodies was added. The plate was then incubated for 1 h at 37 °C and after this period, each well was washed three times with 0.05% Tween 20. Then, 50 μL of anti-His diluted 1:2000 was added and incubated for 1 h, the washes repeated and finally, TMB substrate was added to reveal the color (Sigma, T0440). These results were used to determine with which clone the work would proceed.

From the selected clone, several protein inductions were performed following the protocol described above, the protein was obtained from the inclusion bodies and each batch was pooled.

For the protein purification, 4 mL of a 50% slurry of His60 resin (Clontech (Fremont, CA, USA), 635660) was rinsed with H_2_Odd water and equilibrated in 8 M urea, 50 mM Tris-HCl, pH 8. Soluble inclusion bodies obtained from various batches were added to the resin in a 50 mL tube, and the tube was rotated on a wheel at room temperature for 1 h. The resin was centrifuged for 5 min at 1000 rpm at room temperature and added to a column. The column was washed with 30 mL of 8 M urea and 50 mM Tris-HCl, pH 8 (wash buffer). The vNAR was eluted with 10 mL of increasing concentrations of imidazole in wash buffer (10, 50, 75, 100, 150, and 200 mM). All purification steps were performed at room temperature.

### 4.3. Refolding of V13 Protein and Western Blot

Fractions were analyzed by 20% SDS-PAGE at ~5 μg/well. Pure fractions were pooled and stored at 4 °C for up to 1 week or frozen at −20 °C for longer periods. The vNAR was refolded as follows: purified vNAR in elution buffer was supplemented with fresh 10 mM β-ME. The tubes were then incubated on the benchtop for 30 min, rapidly diluted and mixed with refolding buffer (50 mM Tris-HCl, 0.1 M NaCl, pH 8.0, 2 mM glutathione (GSH), 0.4 mM glutathione disulfide (GSSG)) to a final concentration of 50 μg/mL, and incubated at room temperature for an additional 16 h without shaking. The refolded vNAR was concentrated on His60 resin (~0.5–1 mL) and eluted with 200 mM imidazole and 50 mM Tris-HCl, pH 8. The eluate was concentrated on Amicon filters (3 kDa cutoff, Merck Millipore (Burlington, MA, USA), ACS500324), and the buffer was exchanged on a filter or by dialysis (6 kDa cutoff, Merck Millipore, UFC 900324) against 25 mM Na_2_HPO_4_, pH 7.8, 0.1 M NaCl, 1 mM EDTA buffer overnight at 4 °C. The bacteria endotoxins were removed using Detoxi-Gel Endotoxin Removing Gel (Thermo Scientific (Waltham, MA, USA), 20339) and finally, the quantification of residual endotoxins was determined with the Limulus Amebocyte Lysate kit (Lonza (Basel, Switzerland), QCL-1000).

For the Western blot, the gel was transferred to a PVDF membrane on a semidry system at 200 mA for 1 h. The membrane was then incubated with 5% skim milk for 1 h with agitation at room temperature. V13 was detected with anti-HA-HRP (Roche (Basel, Switzerland), 11965085001), diluted 1:1000 in 3% skimmed milk and incubated for 1 h at room temperature with continuous shaking. The membrane was washed 3 times with 1× PBS/0.05% Tween (PBST-0.05) for 3 min each. Finally, the signals were detected after 5, 10, and 30 s of incubation with ECL substrate (Thermo Scientific Pierce, 167 32106).

### 4.4. Co-Culture Angiogenesis Assay

We evaluated the vNAR in a co-culture assay with NHDFs and GFP-labeled HUVECs cells using the CellPlayer™ GFP-AngioKit (EssenBio, Ann Arbor, MI, USA, 4507). This initial culture medium was replaced by Essen BioScience 96-well Optimized Medium after incubation on days 4, 5, 7, 10, and 14.

Over these days, the vNARs were added to the wells at various concentrations (0.59, 1.17, 2.34, 4.69, 9.38, 18.75, 37.5, 75, 150, and 300 μg/mL) in the presence of 4 ng/mL VEGF_165_, and the results were compared with wells that lacked vNAR V13 and contained only 4 ng/mL VEGF_165_ as a positive control for angiogenesis and also was compared with wells that contained 20 μM suramin and 4 ng/mL VEGF_165_ as negative control (Cell Player Angiogenesis Stem Kit VEGF/Suramin Supplement kit, EssenBio Science, 4509). vNAR treatments were performed in quadruplicate, with 6 images taken per well. The tube length (mm/mm^2^) and inhibition of branch points (1/mm^2^) in all experimental conditions were measured and analyzed in total of 336 h. The co-culture assay results were analyzed by the non-parametric statistical Friedman test and Bonferroni post hoc test, compared with the 4 ng/mL VEGF_165_ control (see supplemental materials). Concentration-response curves were generated using a non-linear regression model to determine the IC_50_ values of vNAR V13 for tube length and branch point inhibition.

### 4.5. Ethics Statements

This study was carried out in accordance with the Mexican guidelines NOM-062-ZOO-1999. The protocol was approved for the Institutional Committee for the Care and Use of Laboratory Animals (Chemistry Faculty, UNAM, Mexico City, Mexico), approval number 089/14, date 09/11/2014) and all efforts were made to minimize animal suffering and reduce the number of animals per assay. The animals were housed under standard laboratory conditions with food and water ad libitum.

### 4.6. Oxygen-Induced Retinopathy (OIR) Model

To evaluate the in vivo efficacy of V13 against VEGF_165_, we used an oxygen-induced retinopathy (OIR) mouse model which is widely described in Stahl [50].

Forty neonatal C57BL/6:Hsd mice from UNAM-Harlan Center were separated into 2 groups of 20 mice each, and then the mice were exposed to hyperoxic conditions (75% oxygen); this environment obliterates the capillaries in the retina. Then, mice were placed in a normoxic environment (21% oxygen) and one group received eye drops 0.1 μg/mL of V13 and the other group received placebo eye drops every 6 h for 7 days. The placebo comprised sterile saline solution for ophthalmic use. After the last treatment application, animals were euthanized by an intraperitoneal injection with pentobarbital and then, both eyes of each mouse were enucleated and examined by immunohistology to determine the level of neovascularization in V13-treated versus placebo-treated retinas. The number of neovascular cells in the periphery of the ocular tissue was counted by histopathology. The enucleated eyes were flat mounted on microscope slides and then, evaluated by microscopy using routine stains (hematoxylin/eosin and periodic acid-Schiff PAS) and, the endothelial cells between the ganglion cell layer and the adjacent layer were counted. The total number of endothelial cells was calculated in 10 high power fields (×40); endothelial cells that were attached to the posterior capsule of the lens (remnants of the tunica vasculosa lentis) and those in other layers of the sensory retina were not included [51]. An unpaired (two sample) *T*-test was applied.

### 4.7. Intraocular Penetration of V13 by ELISA

To establish a background, we made a first approach by examining the penetration of the vNAR, administrating topically a solution of V13 vNAR in one New Zealand adult rabbit eye (total body weight between 2 kg and 2.5 kg) every 20 min for 10 h at 0.1 µg/mL in 1× PBS and, measuring its penetration after 3, 5, 8, 10, and 24 h. The eye drops were administered at the center of the corneal surface. Samples of aqueous humor from one eye were taken at each time point for further analysis. Time 0 corresponded to the value in an untreated rabbit and was used to normalize the ELISA results. All surgical procedures were performed under general anesthesia. To determine the V13 vNAR concentrations in the aqueous humor, microtiter ELISA plates were coated with 50 ng/well of rhVEGF_165_ for 12 h at 4 °C and washed 3 times with wash buffer (PBST-0.1). The plates were blocked with Stabil Guard Immunoassay Stabilizer (BSA-Free) (Surmodics, (Eden Prairie, MN, USA), SG01-1000) for 1 h at room temperature (RT). A total of 100 μL of aqueous humor samples was loaded and incubated for 2 h at RT. The plates were washed 5 times with wash buffer. Anti-HA tag secondary antibody (Genscript (Piscataway, NJ, USA), A01244-100) was diluted in wash buffer to 50 ng/mL and incubated for 1.5 h in RT (100 μL/well). The plates were washed as above. Next, streptavidin peroxidase (1:150,000 in PBST-0.5 buffer) was added, and the plate was incubated for 30 min at RT. After 5 washes, the reaction was visualized with 100 μL/well of chromogenic TMB substrate (Fitzgerald, (Acton, MA, USA), 85R-122), incubated for 30 min. The reaction was stopped with 100 μL 0.05 N HCl, and the absorbance was measured on an ELISA microplate reader (Bio-Rad, Hercules, CA, USA) at 450 nm. For reference, we generated standard curves of 1:2 serial dilutions of V13 vNAR, from 100 ng to 0.097 ng/mL. All dilutions were analyzed at 211 h of incubation. As a negative control, we used PBST-0.5.

## 5. Conclusions

Our present study provided initial evidence that the V13 selected against the VEGF_165_ cytokine could recognize it in an ELISA assay and could neutralize angiogenesis in vitro, giving as a result a diminished tube and branch point formation abilities compared with the suramin control.

In addition, the V13 vNAR anti-angiogenesis capacity resulted in an endothelial cell count diminution in the OIR mouse model and more importantly, we demonstrated for the first time the penetration of a vNAR in a healthy rabbit eye. Considering the unique features of this antibody fragment and our findings in the eye model, we suggest the possibility for the use of V13 in other pathologies where the VEGF_165_ is overproduced, such as neovascular types of cancer.

## 6. Patents

The content of this research article is protected by US8496933B2 and US 9399677B2. 

## Supplementary Materials

The following are available online at http://www.mdpi.com/1660-3397/16/4/113/s1, Table I: All the diluted samples were analyzed at 211 h of incubation in the angiogenesis co-culture assays, because in this point the response detected is constant. Statistical significance was determined using Two-Way ANOVA analysis with a Bonferroni post-hoc analysis comparing to the 4 ng/mL VEGF control. Levels of significance are: * *p* < 0.05, ** *p* < 0.01 and *** *p* < 0.001.

## References

[1] Boyer D., Hopkins J., Sorof J., Ehrlich J. (2013). Anti-vascular endothelial growth factor therapy for diabetic macular edema. Ther. Adv. Endocrinol. Metab..

[2] Ferrara N., Kerbel R.S. (2005). Angiogenesis as a therapeutic target. Nature.

[3] Miao H.Q., Hu K., Jimenez X., Navarro E., Zhang H., Lu D., Ludwig D.L., Balderes P., Zhu Z. (2006). Potent neutralization of VEGF biological activities with a fully human antibody Fab fragment directed against VEGF receptor 2. Biochem. Biophys. Res. Commun..

[4] Amadio M.L., Govoni S., Pascale A. (2016). Targeting VEGF in eye neovascularization: what’s new?: a comprehensive review on current therapies and oligonucleotide-based interventions under development. Pharmacol. Res..

[5] Schmidt-Erfurth U., Chong V., Loewenstein A., Larsen M., Souied E., Schlingemann R., Eldem B., Monés J., Richard G., Bandello F. (2014). Guidelines for the management of neovascular age-related macular degeneration by the European Society of Retina Specialists (EURETINA). Br. J. Ophthalmol..

[6] Amadio M., Bucolo C., Leggio G.M., Drago F., Govoni S., Pascale A. (2010). The PKCbeta/HuR/VEGF pathway in diabetic retinopathy. Biochem. Pharmacol..

[7] Lupo G., Motta C., Giurdanella G., Anfuso C.D., Alberghina M., Drago F., Salomone S., Bucolo C. (2013). Role of phospholipases A2 in diabetic retinopathy: In vitro and in vivo studies. Biochem. Pharmacol..

[8] El-Asrar A.M.A. (2012). Role of inflammation in the pathogenesis of diabetic retinopathy. Middle East Afr. J. Ophthalmol..

[9] Hanout M., Ferraz D., Ansari M., Maqsood N., Kherani S., Sepah Y.J., Rajagopalan N., Ibrahim M., Do D.V., Nguyen Q.N. (2013). Therapies for neovascular age-related macular degeneration: Current approaches and pharmacologic agents in development. BioMed Res. Int..

[10] Holz F.G., Schmitz-Valckenberg S., Fleckenstein M. (2014). Recent developments in the treatment of age-related macular degeneration. J. Clin. Investig..

[11] Bucolo C., Drago F., Salomone S. (2012). Ocular drug delivery: A clue from nanotechnology. Front. Pharmacol..

[12] Rodrigues E.B., Farah M.E., Maia M., Penha F.M., Regatieri C., Melo G.B., Pinheiro M.M., Zanetti C.R. (2009). Therapeutic monoclonal antibodies in ophthalmology. Prog. Retin. Eye Res..

[13] Ng E.W.M., Adamis A.P. (2005). Targeting angiogenesis, the underlying disorder in neovascular age-related macular degeneration. Can. J. Ophthalmol..

[14] Nguyen D., Luo J., Zhang K., Zhang M. (2013). Current therapeutic approaches in neovascular age-related macular degeneration. Discov. Med..

[15] Michaelides M., Kaines A., Hamilton R.D., Fraser-Bell S., Rajendram R., Quhill F., Boos C.J., Xing W., Egan C., Peto T. (2010). A prospective randomized trial of intravitreal bevacizumab or laser therapy in the management of diabetic macular edema (BOLT study) 12-month data: Report 2. Ophthalmology.

[16] Thomas B.J., Shienbaum G., Boyer D.S., Flynn H.W. (2013). Evolving strategies in the management of diabetic macular edema: Clinical trials and current management. Can. J. Ophthalmol..

[17] Georgopoulos M., Polak K., Prager F., Prünte C., Schmidt-Erfurth U. (2009). Characteristics of severe intraocular inflammation following intravitreal injection of bevacizumab (Avastin). Br. J. Ophthalmol..

[18] Johnson D., Hollands H., Hollands S., Sharma S. (2010). Incidence and characteristics of acute intraocular inflammation after intravitreal injection of bevacizumab: A retrospective cohort study. Can. J. Ophthalmol..

[19] Wang F., Yu S., Liu K., Chen F.E., Song Z., Zhang X., Xu X., Sun X. (2013). Acute intraocular inflammation caused by endotoxin after intravitreal injection of counterfeit bevacizumab in Shanghai, China. Ophthalmology.

[20] Meyer C.H., Holz F.G. (2011). Preclinical aspects of anti-VEGF agents for the treatment of wet AMD: Ranibizumab and bevacizumab. Eye.

[21] Semeraro F., Morescalchi F., Duse S., Gambicorti E., Romano M.R., Costagliola C. (2014). Systemic thromboembolic adverse events in patients treated with intravitreal anti-VEGF drugs for neovascular age-related macular degeneration: An overview. Expert Opin. Drug Saf..

[22] Kovaleva M., Ferguson L., Steven J., Porter A., Barelle C. (2014). Shark variable new antigen receptor biologics a novel technology platform for therapeutic drug development. Expert Opin. Biol. Ther..

[23] Zielonka S., Empting M., Grzeschik J., Könning D., Barelle C.J., Kolmar H. (2015). Structural insights and biomedical potential of IgNAR scaffolds from sharks. mAbs.

[24] Hudson P., Souriau C. (2003). Engineered antibodies. Nat. Med..

[25] Wesolowski J., Alzogaray V., Reyelt J., Unger M., Juarez K., Urrutia M., Cauerhff A., Danquah W., Rissiek B., Scheuplein F. (2009). Single domain antibodies: Promising experimental and therapeutic tools in infection and immunity. Med. Microbiol. Immunol..

[26] Müller M.R., Saunders K., Grace C., Jin M., Piche-Nicholas M., Steven J., O’Dwyer R., Wu L., Khetemenee L., Vugmeyster Y. (2012). Improving the pharmacokinetic properties of biologics by fusion to an anti-HSA shark vNAR domain. mAbs.

[27] Bojalil R., Mata-González M.T., Sánchez-Muñoz F., Yee Y., Argueta I., Bolaños L., Amezcua-Guerra L.M., Camacho-Villegas T.A., Sánchez-Castrejón E., García-Ubbelohde W.J. (2013). Anti-tumor necrosis factor VNAR single domains reduce lethality and regulate underlying inflammatory response in a murine model of endotoxic shock. BMC Immunol..

[28] Kopsidas G., Roberts A.S., Coia G., Streltsov V.A., Nuttall S.D. (2006). In vitro improvement of a shark IgNAR antibody by Qb replicase mutation and ribosome display mimics in vivo affinity maturation. Immunol. Lett..

[29] Griffiths K., Dolezal O., Parisi K., Angerosa J., Dogovski C., Barraclough M., Sanalla M., Casey J.-L., González I., Perugini M.A. (2013). Shark variable new antigen receptor (vNAR) single domain antibody fragments: Stability and diagnostic applications. Antibodies.

[30] Liu J.L., Anderson G.P., Goldman E.R. (2007). Isolation of antitoxin single domain antibodies from a semi-synthetic spiny dogfish shark display library. BMC Biotechnol..

[31] Streltsov V.A., Varghese J.N., Carmichael J.A., Irving R.A., Hudson P.J., Nuttall S.D. (2004). Structural evidence for evolution of shark Ig new antigen receptor variable domain antibodies from a cell-surface receptor. Proc. Natl. Acad. Sci. USA.

[32] Liu J.L., Anderson G.P., Delehanty J.B., Baumann R., Hayhurst A., Goldman E.R. (2007). Selection of cholera toxin specific IgNAR single-domain antibodies from a naive shark library. Mol. Immunol..

[33] Zielonka S., Weber N., Becker S., Doerner A., Christmann A., Christmann C., Uth C., Fritz J., Schäfer E., Steinmann B. (2014). Shark attack: High affinity binding proteins derived from shark vNAR domains by stepwise in vitro affinity maturation. J. Biotechnol..

[34] Geoghegan K.F., Dixon H.B.F., Rosner P.J., Hoth L.R., Lanzetti A.J., Borzilleri K.A., Marr E.S., Pezzullo L.H., Martin L.B., LeMotte P.K. (1999). Spontaneous a-*N*-6-Phosphogluconoylation of a “His Tag” in *Escherichia coli*: The cause of extra mass of 258 or 178 Da in fusion proteins. Anal. Biochem..

[35] Waltenberger J., Mayr U., Frank H., Hombach V. (1996). Suramin is a potent inhibitor of vascular endothelial growth factor: A contribution to the molecular basis of its antiangiogenic action. J. Mol. Cell. Cardiol..

[36] Papadopoulos N., Martin J., Ruan Q., Rafique A., Rosconia M.P., Shi E., Pyles E.A., Yancopoulos G.D., Stahl N., Wiegard S.J. (2012). Binding and neutralization of vascular endothelial growth factor (VEGF) and related ligands by VEGF Trap, ranibizumab and bevacizumab. Angiogenesis.

[37] Dastjerdi M.H., Sadrai Z., Saban D.R., Zhang Q., Dana R. (2011). Corneal penetration of topical and subconjunctival bevacizumab. Investig. Ophthalmol. Vis. Sci..

[38] Zhang S.X., Ma J.X. (2007). Ocular neovascularization: Implication of endogenous angiogenesis inhibitor and potential therapy. Prog. Retin. Eye Res..

[39] Falavarjani K.G., Nguyen Q.D. (2013). Adverse events and complications associated with intravitreal injection of anti-VEGF agents: A review of literature. Eye.

[40] Ferrara N., Adamis A.P. (2016). Ten years of anti-vascular endothelial growth factor therapy. Nat. Rev. Drug Discov..

[41] Agrahari V., Agrahari V., Mandal A., Pal D., Mitra A.K. (2016). How are we improving the delivery to back of the eye? advances and challenges of novel therapeutic approaches. Expert Opin. Drug Deliv..

[42] Pecen P.E., Kaiser P.K. (2015). Current phase 1/2 research for neovascular age-related macular degeneration. Curr. Opin. Ophthalmol..

[43] Fleetwood F., Güler R., Gordon E., Ståhl S., Claesson-Welsh L., Löfblom J. (2015). Novel affinity binders for neutralization of vascular endothelial growth factor (VEGF) signaling. Cell. Mol. Life Sci..

[44] Stevenson W., Cheng S.-F., Dastjerdi M.H., Ferrari G., Reza D. (2012). Corneal neovascularization and the utility of topical VEGF inhibition: Ranibizumab (Lucentis) vs. bevacizumab (Avastin). Ocul. Surf..

[45] Goel M., Picciani R.G., Lee R.K., Bhattacharya S.K. (2010). Aqueous humor dynamics: A review. Open Ophthalmol. J..

[46] Chen J.J., Ebmeier S.E., Sutherland W.M., Ghazi N.G. (2011). Potential penetration of topical ranibizumab (Lucentis) in the rabbit eye. Eye.

[47] Kovaleva M., Johnson K., Steven J., Barelle C.J., Porter A. (2017). Therapeutic potential of shark anti-ICOSL vNAR domains is exemplified in a murine model of autoimmune non-infectious uveitis. Front. Immunol..

[48] Camacho-Villegas T.A., Mata-Gonzalez M.T., Paniagua-Solis J., Sanchez E., Licea A. (2013). Human TNF cytokine neutralization with a vNAR from *Heterodontus francisci* shark: A potential therapeutic use. mAbs.

[49] Barbas C.F., Burton D.R., Scott J.K. (2001). Phage Display: A Laboratory Manual.

[50] Stahl A., Connor K.M., Sapieha P., Chen J., Dennison R.J., Krah N.M., Seaward M.R., Willett K.L., Aderman C.M., Guerin K.I. (2010). The mouse retina as an angiogenesis model. Investig. Ophthalmol. Vis. Sci..

[51] Smith L.E.H., Wesoloiuski E., McLellan A., Kostyk S.K., D’Amato X.R., Sullivan R., D’Amore P.A. (1994). Oxygen-induced retinopathy in the mouse. Investig. Ophthalmol. Vis. Sci..

