# Supplementary material for: Intraocular Penetration of a vNAR: In Vivo and In Vitro VEGF165 Neutralization
Source: Mar Drugs. 2018 Mar 31;16(4):113. doi: 10.3390/md16040113 (PMC5923400; doi:10.3390/md16040113)
Supplement: Supplementary file 1 [file marinedrugs-16-00113-s001.pdf]

marine drugs

Supplementary Material

In vivo and *in vitro* $VEGF_{165}$ Neutralization via Intraocular Penetration of a vNAR

Tanya A. Camacho-Villegas[1,2], María Teresa Mata-González[3], Walter García-Ubbelohd[3], Linda Núñez-García[3], Carolina Elosua[4], Jorge F. Paniagua-Solis[3] and Alexei Licea[2*]

Table I. All the diluted samples were analyzed at 211 h of incubation in the angiogenesis co-culture assays, because in this point the response detected is constant. Statistical significance was determined using Two-Way ANOVA analysis with a Bonferroni post-hoc analysis comparing to the 4 ng/mL VEGF control. Levels of significance are: * $p<0.05$, ** $p<0.01$ and *** $p<0.001$.

Time (h)	4 ng/mL VEGF vs untreated cells				VEGF + 4.69 µg/mL vNAR				VEGF + 9.38 µg/mL vNAR				VEGF + 18.75 µg/mL vNAR				VEGF + 37.57 µg/mL vNAR				VEGF + 75 µg/mL vNAR				VEGF + 150 µg/mL vNAR				VEGF + 300 µg/mL vNAR			
	Tube length		Branch point		Tube length		Branch point		Tube length		Branch point		Tube length		Branch point		Tube length		Branch point		Tube length		Branch point		Tube length		Branch point		Tube length		Branch point	
Elapsed	p-value	s	p-value	s	p-value	s	p-value	s	p-value	s	p-value	s	p-value	s	p-value	s	p-value	s	p-value	s	p-value	s	p-value	s	p-value	s	p-value	s	p-value	s	p-value	s
0	P > 0.05	ns	P > 0.05	ns	P > 0.05	ns	P > 0.05	ns	P > 0.05	ns	P > 0.05	ns	P > 0.05	ns	P > 0.05	ns	P > 0.05	ns	P > 0.05	ns	P > 0.05	ns	P > 0.05	ns	P > 0.05	ns	P > 0.05	ns	P > 0.05	ns	P > 0.05	ns
11	P > 0.05	ns	P > 0.05	ns	P > 0.05	ns	P > 0.05	ns	P > 0.05	ns	P > 0.05	ns	P > 0.05	ns	P > 0.05	ns	P > 0.05	ns	P > 0.05	ns	P > 0.05	ns	P > 0.05	ns	P > 0.05	ns	P > 0.05	ns	P > 0.05	ns	P > 0.05	ns
23	P > 0.05	ns	P > 0.05	ns	P > 0.05	ns	P > 0.05	ns	P > 0.05	ns	P > 0.05	ns	P > 0.05	ns	P > 0.05	ns	P > 0.05	ns	P > 0.05	ns	P > 0.05	ns	P > 0.05	ns	P > 0.05	ns	P > 0.05	ns	P > 0.05	ns	P > 0.05	ns
35	P > 0.05	ns	P > 0.05	ns	P > 0.05	ns	P > 0.05	ns	P > 0.05	ns	P > 0.05	ns	P > 0.05	ns	P > 0.05	ns	P > 0.05	ns	P > 0.05	ns	P > 0.05	ns	P > 0.05	ns	P > 0.05	ns	P > 0.05	ns	P > 0.05	ns	P > 0.05	ns
47	P > 0.05	ns	P > 0.05	ns	P > 0.05	ns	P > 0.05	ns	P > 0.05	ns	P > 0.05	ns	P > 0.05	ns	P > 0.05	ns	P > 0.05	ns	P > 0.05	ns	P > 0.05	ns	P > 0.05	ns	P > 0.05	ns	P > 0.05	ns	P > 0.05	ns	P > 0.05	ns
59	P > 0.05	ns	P > 0.05	ns	P > 0.05	ns	P > 0.05	ns	P > 0.05	ns	P > 0.05	ns	P > 0.05	ns	P > 0.05	ns	P > 0.05	ns	P > 0.05	ns	P > 0.05	ns	P > 0.05	ns	P > 0.05	ns	P > 0.05	ns	P > 0.05	ns	P > 0.05	ns
71	P > 0.05	ns	P > 0.05	ns	P > 0.05	ns	P > 0.05	ns	P > 0.05	ns	P > 0.05	ns	P > 0.05	ns	P > 0.05	ns	P > 0.05	ns	P > 0.05	ns	P > 0.05	ns	P > 0.05	ns	P > 0.05	ns	P > 0.05	ns	P > 0.05	ns	P > 0.05	ns
81	P > 0.05	ns	P > 0.05	ns	P > 0.05	ns	P > 0.05	ns	P > 0.05	ns	P > 0.05	ns	P > 0.05	ns	P > 0.05	ns	P > 0.05	ns	P > 0.05	ns	P > 0.05	ns	P > 0.05	ns	P > 0.05	ns	P > 0.05	ns	P > 0.05	ns	P > 0.05	ns
93	P > 0.05	ns	P > 0.05	ns	P > 0.05	ns	P > 0.05	ns	P > 0.05	ns	P > 0.05	ns	P > 0.05	ns	P > 0.05	ns	P > 0.05	ns	P > 0.05	ns	P > 0.05	ns	P > 0.05	ns	P > 0.05	ns	P > 0.05	ns	P > 0.05	ns	P > 0.05	ns
105	P < 0.05	*	P > 0.05	ns	P > 0.05	ns	P > 0.05	ns	P > 0.05	ns	P > 0.05	ns	P > 0.05	ns	P > 0.05	ns	P > 0.05	ns	P > 0.05	ns	P > 0.05	ns	P > 0.05	ns	P<0.001	***	P > 0.05	ns	P<0.001	***	P > 0.05	ns
117	P<0.001	***	P<0.001	***	P > 0.05	ns	P > 0.05	ns	P > 0.05	ns	P > 0.05	ns	P > 0.05	ns	P > 0.05	ns	P<0.01	**	P < 0.05	*	P<0.001	***	P<0.001	***	P<0.001	***	P<0.001	***	P<0.001	***	P<0.001	***
129	P<0.001	***	P<0.001	***	P > 0.05	ns	P > 0.05	ns	P > 0.05	ns	P > 0.05	ns	P > 0.05	ns	P > 0.05	ns	P<0.001	***	P<0.001	***	P<0.001	***	P<0.001	***	P<0.001	***	P<0.001	***	P<0.001	***	P<0.001	***
141	P<0.001	***	P<0.001	***	P > 0.05	ns	P > 0.05	ns	P > 0.05	ns	P < 0.05	*	P<0.001	***	P<0.001	***	P<0.001	***	P<0.001	***	P<0.001	***	P<0.001	***	P<0.001	***	P<0.001	***	P<0.001	***	P<0.001	***
153	P<0.001	***	P<0.001	***	P > 0.05	ns	P > 0.05	ns	P > 0.05	ns	P<0.001	***	P<0.001	***	P<0.001	***	P<0.001	***	P<0.001	***	P<0.001	***	P<0.001	***	P<0.001	***	P<0.001	***	P<0.001	***	P<0.001	***
165	P<0.001	***	P<0.001	***	P > 0.05	ns	P > 0.05	ns	P<0.001	***	P<0.001	***	P<0.001	***	P<0.001	***	P<0.001	***	P<0.001	***	P<0.001	***	P<0.001	***	P<0.001	***	P<0.001	***	P<0.001	***	P<0.001	***
176	P<0.001	***	P<0.001	***	P > 0.05	ns	P > 0.05	ns	P<0.001	***	P<0.001	***	P<0.001	***	P<0.001	***	P<0.001	***	P<0.001	***	P<0.001	***	P<0.001	***	P<0.001	***	P<0.001	***	P<0.001	***	P<0.001	***
188	P<0.001	***	P<0.001	***	P > 0.05	ns	P > 0.05	ns	P<0.001	***	P<0.001	***	P<0.001	***	P<0.001	***	P<0.001	***	P<0.001	***	P<0.001	***	P<0.001	***	P<0.001	***	P<0.001	***	P<0.001	***	P<0.001	***
**200**	**P<0.001**	*******	**P<0.001**	*******	P > 0.05	ns	P > 0.05	ns	P<0.001	***	P<0.001	***	P<0.001	***	P<0.001	***	P<0.001	***	P<0.001	***	P<0.001	***	P<0.001	***	P<0.001	***	P<0.001	***	P<0.001	***	P<0.001	***
212	P<0.001	***	P<0.001	***	P > 0.05	ns	P > 0.05	ns	P<0.001	***	P<0.001	***	P<0.001	***	P<0.001	***	P<0.001	***	P<0.001	***	P<0.001	***	P<0.001	***	P<0.001	***	P<0.001	***	P<0.001	***	P<0.001	***
224	P<0.001	***	P<0.001	***	P > 0.05	ns	P > 0.05	ns	P<0.001	***	P<0.001	***	P<0.001	***	P<0.001	***	P<0.001	***	P<0.001	***	P<0.001	***	P<0.001	***	P<0.001	***	P<0.001	***	P<0.001	***	P<0.001	***
236	P<0.001	***	P<0.001	***	P > 0.05	ns	P > 0.05	ns	P<0.001	***	P<0.001	***	P<0.001	***	P<0.001	***	P<0.001	***	P<0.001	***	P<0.001	***	P<0.001	***	P<0.001	***	P<0.001	***	P<0.001	***	P<0.001	***
247	P<0.001	***	P<0.001	***	P > 0.05	ns	P > 0.05	ns	P<0.001	***	P<0.001	***	P<0.001	***	P<0.001	***	P<0.001	***	P<0.001	***	P<0.001	***	P<0.001	***	P<0.001	***	P<0.001	***	P<0.001	***	P<0.001	***
259	P<0.001	***	P<0.001	***	P > 0.05	ns	P > 0.05	ns	P<0.001	***	P<0.001	***	P<0.001	***	P<0.001	***	P<0.001	***	P<0.001	***	P<0.001	***	P<0.001	***	P<0.001	***	P<0.001	***	P<0.001	***	P<0.001	***
271	P<0.001	***	P<0.001	***	P > 0.05	ns	P > 0.05	ns	P<0.001	***	P<0.001	***	P<0.001	***	P<0.001	***	P<0.001	***	P<0.001	***	P<0.001	***	P<0.001	***	P<0.001	***	P<0.001	***	P<0.001	***	P<0.001	***
283	P<0.001	***	P<0.001	***	P > 0.05	ns	P > 0.05	ns	P<0.001	***	P<0.001	***	P<0.001	***	P<0.001	***	P<0.001	***	P<0.001	***	P<0.001	***	P<0.001	***	P<0.001	***	P<0.001	***	P<0.001	***	P<0.001	***
295	P<0.001	***	P<0.001	***	P<0.01	**	P < 0.05	*	P<0.001	***	P<0.001	***	P<0.001	***	P<0.001	***	P<0.001	***	P<0.001	***	P<0.001	***	P<0.001	***	P<0.001	***	P<0.001	***	P<0.001	***	P<0.001	***
307	P<0.001	***	P<0.001	***	P<0.01	**	P > 0.05	ns	P<0.001	***	P<0.001	***	P<0.001	***	P<0.001	***	P<0.001	***	P<0.001	***	P<0.001	***	P<0.001	***	P<0.001	***	P<0.001	***	P<0.001	***	P<0.001	***
319	P<0.001	***	P<0.001	***	P<0.01	**	P > 0.05	ns	P<0.001	***	P<0.001	***	P<0.001	***	P<0.001	***	P<0.001	***	P<0.001	***	P<0.001	***	P<0.001	***	P<0.001	***	P<0.001	***	P<0.001	***	P<0.001	***
